# SHIP-1 Regulates Phagocytosis and M2 Polarization Through the PI3K/Akt–STAT5–Trib1 Circuit in *Pseudomonas aeruginosa* Infection

**DOI:** 10.3389/fimmu.2020.00307

**Published:** 2020-03-18

**Authors:** Shugang Qin, Jiaxin Li, Chuanmin Zhou, Breanna Privratsky, Jacob Schettler, Xin Deng, Zhenwei Xia, Yong Zeng, Hong Wu, Min Wu

**Affiliations:** ^1^Department of Biomedical Sciences, School of Medicine and Health Sciences, University of North Dakota, Grand Forks, ND, United States; ^2^State Key Laboratory of Biotherapy, West China Hospital, Sichuan University, Chengdu, China; ^3^Department of Liver Surgery, West China Hospital, Sichuan University, Chengdu, China; ^4^Department of Pediatrics and Department of Pulmonary & Critical Care Medicine, Ruijin Hospital Affiliated to Shanghai Jiao Tong University School of Medicine, Shanghai, China

**Keywords:** *Pseudomonas aeruginosa*, SHIP-1, PI3K, Akt, STAT5, M1 macrophages, M2 macrophage

## Abstract

SHIP-1 is an inositol phosphatase that hydrolyzes phosphatidylinositol 3-kinase (PI3K) products and negatively regulates protein kinase B (Akt) activity, thereby modulating a variety of cellular processes in mammals. However, the role of SHIP-1 in bacterial-induced sepsis is largely unknown. Here, we show that SHIP-1 regulates inflammatory responses during Gram-negative bacterium *Pseudomonas aeruginosa* infection. We found that infected-SHIP-1^−/−^ mice exhibited decreased survival rates, increased inflammatory responses, and susceptibility owing to elevated expression of PI3K than wild-type (WT) mice. Inhibiting SHIP-1 *via* siRNA silencing resulted in lipid raft aggregates, aggravated oxidative damage, and bacterial burden in macrophages after PAO1 infection. Mechanistically, SHIP-1 deficiency augmented phosphorylation of PI3K and nuclear transcription of signal transducer and activator of transcription 5 (STAT5) to induce the expression of Trib1, which is critical for differentiation of M2 but not M1 macrophages. These findings reveal a previously unrecognized role of SHIP-1 in inflammatory responses and macrophage homeostasis during *P. aeruginosa* infection through a PI3K/Akt–STAT5–Trib1 axis.

## Introduction

The Src homology 2-containing inositol 5-phosphatase, SHIP-1, is a 145-kDa protein highly expressed in hematopoietic cells (1). SHIP-1 negatively regulates phosphatidylinositol 3-kinase (PI3K) activity through hydrolysis of the 5′-phosphate from the PI3K lipid product phosphatidylinositol 3,4,5-triphosphate [PI (3,4,5) P3] to phosphatidylinositol (3,4)-bisphosphate (PI (3,4) P2) (2). The PI3K–Akt pathway affects a spectrum of cellular functions including metabolism, growth, proliferation, survival, and adhesion (3). Mast cells and macrophages, which are essential for the negative regulation of type II immune responses to drive lung spontaneous inflammation and injury, are over-activated in SHIP-1^−/−^ mice after *Candida albicans* infection (4). Pulmonary inflammation and M2-polarized macrophages were greatly exacerbated in SHIP-1^−/−^ BALB/c mice (5). Other studies have also found that bacteria utilize lipid rafts to evade immune defense, indicating complex and intertwined signaling circuits in host defense (6, 7). Evaluating interactions between SHIP-1 and lipid rafts may help to understand their role in innate immunity during bacterial infection.

Macrophages, as an important component of the immune system to phagocytize and clear pathogens, are related to the control of inflammation (8). Macrophages are mainly composed of two functional subtypes, M1 and M2 (8, 9). Classically activated macrophages (M1) play a major role in defense against bacteria and viruses by activating inflammatory signaling pathways through secreting pro-inflammatory cytokines (IFN-γ, IL-1, IL-6, IL-12, IL-23, and TNFα) (10). Alternatively activated macrophages (M2) are activated by IL-4 and IL-13 and participate in tissue remodeling and fibrosis by regulation of anti-inflammatory response signal production through secreting IL-10 and TGF-β (11, 12). M2/M1 macrophage polarization is essential for maintaining macrophage phagocytosis and immune function (13, 14). Signal transducer and activator of transcription 5 (STAT5), which plays a critical role in the development of the hematopoietic system, can dominate the function of multiple cytokines by regulating the transcription of distinct target genes (15). Studies have shown that increased STAT5 activity in SHIP^−/−^ mice may play a key role in promoting M2 macrophage polarization (16). The M2 phenotype is suppressed when STAT5(^−/−^) BM cells are used in response to IL-3 and granulocyte-macrophage colony-stimulating factor (GM-CSF) stimuli, which can skew murine macrophage progenitors toward an M2 phenotype in the absence of SHIP (17, 18). Tribbles homolog 1 (Trib1), as a member of the mammalian Tribbles homolog pseudokinase family, has been shown to be critical for the differentiation of tissue residence of M2-like macrophages (19). Trib1 induced macrophages to express M2 phenotype markers in prostate cancer (20), whereas Trib1 deficiency attenuated the expression of M2-marker genes upon IL-4 stimulation (21). M2 macrophages exhibited a selective depletion after myocardial infarction in Trib1^−/−^ mice compared with wild-type (WT) mice (22). To date, no studies of the effects of Trib1, SHIP-1, and STAT5 on inflammatory response and subsequent sepsis induced by *Pseudomonas aeruginosa* infection have been reported.

*Pseudomonas aeruginosa*, an opportunistic bacterium, can infect almost any human organs and is particularly prevalent in immunocompromised individuals, such as patients with HIV-1, tuberculosis infection, cancer, and cystic fibrosis (23, 24). Whereas, antibiotic-resistant strains of PAO1 expand at an alarming pace, the development of effective anti-infectious measures including new antibiotics, chemical drugs, and vaccines has been disappointingly slow. SHIP is essential for endotoxin tolerance by suppressing the generation of M1 macrophages (25), suggesting that SHIP-1 is key for lung immune balance and suppression of inflammation disorders by controlling macrophage function. Nevertheless, the underlying mechanism of SHIP-1 for modulating macrophage function and inflammatory response remains largely elusive in bacterial infection. We herein studied the role of SHIP-1 in PAO1-induced cytokine responses and the underlying molecular mechanisms using SHIP-1^−/−^ mice. Our research shows that SHIP-1 deficiency sabotaged phagocytosis by macrophages and M2 polarization by activating phosphorylation of PI3K/Akt and STAT5 to induce the expression of Trib1, which is critical for the differentiation of M2 as opposed to M1 macrophages. These findings reveal that SHIP-1 plays important roles in regulating macrophage differentiation and inflammatory responses following *P. aeruginosa* infection.

## Materials and Methods

### Animals

SHIP-1^−/−^ mice were graciously provided by Dr. Chris Baran of the Ohio State University and were backcrossed with the background C57BL/6N mice for seven generations (26). C57BL/6N mice (6–8 weeks) were purchased from the Harlan Laboratory. Both male and female were used and randomly grouped for assays (age of mice ranged from 6 to 8 weeks, body weight 22 ± 3 g) for both SHIP-1^−/−^ mice and C57BL/6N mice. The mouse experiments have been approved by the University of North Dakota Institutional Animal Care and Use Committee (IACUC) and were performed following National Institutes of Health (NIH) guidelines. Mice were anesthetized using 45 mg/kg of ketamine and instilled intranasally with 2 × 10^7^ colony-forming units (CFUs) of PAO1. The animals were dissected when they were moribund. Thereafter, bronchoalveolar lavage fluid (BALF) was performed, and the trachea and lung were excised for homogenization or inflated with 50% optical coherence tomography (OCT) in saline or 10% formalin fixation in phosphate-buffered saline (PBS) (27).

### Cells

Murine alveolar macrophages (MH-S) cells were obtained from American Type Culture Collection (ATCC, Manassas, VA) and maintained following the manufacturer's instructions (28). Primary mouse alveolar macrophages (AM) cells and BALF were isolated by bronchoalveolar lavage as previously described (29). The AM obtained from lavage was centrifuged, and the precipitation (containing AM) was resuspended in Roswell Park Memorial Institute (RPMI) 1,640 medium containing 10% newborn bovine serum (HyClone Laboratories, Logan, UT), 100 U/ml of penicillin, and 100 g/ml of streptomycin (Life Technologies, Rockville, MD) and incubated in a 5% CO_2_ incubator. For confocal studies, the MH-S cells were seeded on glass-bottomed dishes in order to enhance adherence.

### Bacterial Strains

WT strain PAO1 was a gift from Steve Lory (Department of Molecular Genetics, Harvard Medical School). Green fluorescent protein (GFP)–PAO1 was obtained from Gerald Pier (Channing Laboratory, Harvard Medical School) and Dr. Shawn Lewenza (University of Calgary). Pseudomonas aeruginosa PAO1 Xen 41(PAO1 Xen 41) expressing bioluminescence was bought from PerkinElmer Caliper (Waltham, MA). Previous studies have shown that the virulence of these bacteria is consistent with that of PAO1 (30).

### Infection Experiments

Bacteria were grown overnight in Luria–Bertani (LB) broth at 37°C with shaking. Then, the bacteria were pelleted by centrifugation at 5,000 g and resuspended in 10 ml of fresh LB broth. The mid-logarithmic phase of bacteria is determined by measuring the OD600 nm, and density was adjusted to OD600 = 0.5–0.6 (to ensure the same vitality) (1 OD = 1 × 10^9^ cells/ml). Mammalian cells were counted and were washed once with PBS after overnight culture in RPMI 1,640 medium with 10% fetal bovine serum and changed to serum-free and antibiotic-free media immediately before infection. Cells were infected with 20 times the amount of bacteria (the amount of bacteria/the amount of cells = 20:1) for indicated time points; mice were infected with PAO1 (2 × 10^7^ CFU/mouse) (31).

### Bacterial Burden Assay

BALF obtained from mice was diluted to different gradients in PBS, and lung tissue was homogenized with PBS. They then were spread on LB dishes to evenly distribute bacteria. The dishes were cultured in a 37°C incubator overnight, and colonies were counted. Each experiment was performed in triplicates.

### Cell Transfection

MH-S cells were transfected with SHIP-1 small interference RNA, STAT5 siRNA (sc-29496, Santa Cruz), and TRRB-1 siRNA (sc-154620, Santa Cruz) using Lipofectamine 3,000 reagent (Invitrogen, Carlsbad, CA) in serum-free RPMI 1640 medium following the manufacturer's instructions. The knockout efficiency was detected by western blotting after 48-h transfection.

### Western Blotting

Following transfection and/or infection, the MH-S cells were lysed in radio-immunoprecipitation assay (RIPA) buffer containing a protease inhibitor cocktail (Thermo Fisher, Waltham, MA). The lysates were boiled for 10 min after adding loading dye. Equal amounts of each sample (30 μl) were loaded onto 10% sodium dodecyl sulfate (SDS)–polyacrylamide gel electrophoresis and electrophoresed to resolve proteins. The proteins were then transferred to nitrocellulose membranes (Santa Cruz) and blocked for 2 h at room temperature using 5% non-fat dry milk buffer in TBST. The membranes were then incubated with the first antibodies diluted 1:1,000 in 1% non-fat milk blocking buffer overnight at 4°C. After being washed four times with washing buffer, the membranes were incubated with horseradish peroxidase-conjugated secondary antibody (Amersham, St Louis, MO) diluted 1:2,000 for 2 h at room temperature. Signals were detected using enhanced chemiluminescence detection kit (SuperSignal West Pico, Pierce, Rockford, IL). Mouse monoclonal Abs against GAPDH (sc-29496), β-actin (sc-29496), Trib1 (sc-393536), STAT5 (sc-7442), p-STAT5 (sc-101806), SHIP-1 (sc-8425), NF-κB p50 (SC-372), p-NF-κB p50 (sc-271908), IL-4 (SC-32242), and IL-10 (SC-36585) were bought from Santa Cruz. p-Akt (4046s), Akt (2920s), TNF-α (11948S), IL-6 (12912S), IL-1β (12507S), and arginase-1 (93668) were purchased from Cell Signaling Technologies.

### RNA Isolation and Real-Time Quantitative PCR Analysis

RNA was extracted from lung tissues by using TRIzol reagent (Invitrogen) and were reverse transcribed into DNA by using the reverse transcriptase kits (Invitrogen). For gene detection, real-time quantitative PCR reaction (RT-PCR) was performed using iTaq Universal SYBR Green Supermix (Bio-Rad), and the PCR primers used in the article are listed in Supplemental Table 1.

### Nitroblue Tetrazolium Assay

This assay was used to determine the production of superoxide anion in phagocytic cells. BALF cells were obtained from WT mice and SHIP^−/−^mice, then were grown in a 96-well plate in serum-containing medium at 37°C for 4 h, washed three times with PBS buffer, and subsequently infected with PAO1 for 2 h; and 1 μg/ml of nitroblue tetrazolium (NBT) dye (Sigma) was added to each well. The cells were incubated at 37°C for 1 h or until the color developed. The dye was yellow and gave a blue color formazan product upon reduction by superoxide. The reaction was terminated by adding 100 μl of stop solution (10% DMSO; 10% SDS in 50 mM HEPES buffer). The plate was left at room temperature overnight for complete dissolution of formazan product and read at 560-nm absorbance using a multiscan plate reader to quantify the dye conversion. Triplicates were done for each sample and control. Background was corrected by using blanks containing dye alone (32).

### Quantitative Polymorphonuclear Neutrophils by HEMA-3 Staining

The infiltration level of polymorphonuclear neutrophils (PMNs) in the serum was used to assess the degree of inflammatory response as previously described (33). The animal blood was obtained by cardiac blood sampling on the sacrificed mouse and was stained on the smear by HEMA-3 staining (23-122929, Thermo Fisher), quantified by light microscopy at 400 × final magnification.

### Dihydrodichlorofluorescein Diacetate Assay

Dihydrodichlorofluorescein diacetate (H_2_DCF-DA) dye (Molecular Probes, Carlsbad, CA) does not normally fluoresce but emits green fluorescence upon reaction with cellular superoxides. AM cells were obtained from WT mice and SHIP^−/−^ mice and were grown in a 96-well plate in serum-containing medium at 37°C for 4 h, washed three times with PBS buffer, and subsequently infected with PAO1 for 2 h; and an equal amount of dye was added. The plate fluorescence was measured by fluorescence plate reader (BioTek, Winooski, VT) after 1 h incubation using 485-nm excitation and 528-nm emission filter.

### 3-(4,5-Dimethylthiazol-2-yl)-2,5-diphenyltetrazolium Bromide Assay

The 3-(4,5-dimethylthiazol-2-yl)-2,5-diphenyltetrazolium bromide (MTT) assay was used to assess the viability of cells. Mice were anesthetized using 45 mg/kg of ketamine and instilled intranasally with 2 × 10^7^ CFUs of PAO1 for 12 h. AM cells were obtained from infected mice and processed as above; 1 μg/ml of MTT dye was added to each well. The cells were incubated at 37°C for 1 h or until the color change occurred. The dye was reduced to form a purple formazan product inside living cells. Stop solution was added to dissolve the formazan product and quantified by absorbance measurement at 560 nm using a spectrometer plate reader.

### Myeloperoxidase Assay

The myeloperoxidase (MPO) assay was performed as follows. Mice were anesthetized using 45 mg/kg of ketamine and instilled intranasally with 2 × 10^7^ CFUs of PAO1 for 12 h. Lung tissues were obtained from infected mice and homogenized in 50 mM of hexadecyltrimethylammonium bromide, 50 mM of KH_2_PO_4_, pH 6.0, 0.5 mM of EDTA at 1 ml/100 mg of tissue and centrifuged for 15 min at 12,000 rpm at 4°C. Supernatants were decanted, and 100 μl of reaction buffer (0.167 mg/ml of *O*-dianisidine, 50 mM of KH_2_PO_4_, pH 6.0, and 0.0005% mM H_2_O_2_) was added to 100 μl of sample. Absorbance was read at 460 nm in 2-min intervals. Duplicates were done for each sample and control.

### Lipid Peroxidation Assay

Malondialdehyde is an end product of the lipid peroxidation and was measured using a colorimetric assay (Calbiochem, Billerica, MA) according to the manufacturer's instructions. Mice were anesthetized using 45 mg/kg of ketamine and instilled intranasally with 2 × 10^7^ CFUs of PAO1 for 12 h. Lung tissue was obtained from infected mice and homogenized in 62.5 mM of Tris–HCl (pH 6.8) supplemented with complete mini protease inhibitor (Roche Diagnostics, Indianapolis, IN) in equal protein amounts. Duplicates were done for each sample and control.

### Phagocytosis Assays

Elimination of bacteria depends on many factors such as the phagocytosis of macrophages, which is closely related to macrophage polarization (34). GFP–PAO1 strain was used to determine the phagocytosis after infecting MH-S cell for 2 h and washing three times for PBS to remove the surface attached bacteria. The amounts of bacteria were counted by using bacterial burden assay. The amounts of intracellular bacteria were also determined by immunofluorescence, and cell nuclei were stained with DAPI. Then the amounts of intracellular bacteria (GFP–PAO1) were captured under the confocal microscope (Carl Zeiss, White Plains, NY).

### Measurement of Inflammatory Cytokines

Cytokine concentrations were measured by an ELISA kit (eBioscience Co, Invitrogen, San Diego, CA). TNF-α (88-7324-22), IL-1β (88-7013-22), and IL-6 (BMS603-2) were used to represent inflammatory M1 macrophage markers and IL-10 (88-7105-22), IL-4 (BMS613), and TGF-β1 (BMS608-4) for M2 macrophage markers. Ninety-six-well plates (Corning Costar 9018) were coated with 100 μl/well of capture antibody in coating buffer and incubated overnight at 4°C. Cytokine concentrations were determined with corresponding detection horseradish peroxidase (HRP)-conjugated antibodies. Optical absorbance was read at 450 nm and analyzed (30).

### Immunostaining

MH-S cells were grown either on coverslips in a 24-well plate or in glass-bottomed dishes (MatTek, Ashland, MA). For immunostaining, the cells were fixed in 4% paraformaldehyde and permeabilized with 0.5% Triton X-100 in PBS, then incubated with primary Abs at a 1:500 dilution in PBS for 1 h in the dark at room temperature, and then washed three times with PBS buffer. After incubation with the appropriate fluorescein-conjugated secondary Abs, DAPI (Sigma-Aldrich) was used to stain the nucleus for 10 min in the dark at room temperature. The coverslips were mounted on slides with VECTASHIELD mounting medium. The cholera toxin B (CTB) chain containing fluorescein (C34777, Thermo Fisher) was used to track lipid rafts, and arginase-1 (red, M2 type macrophage marker) (93668, CST) and TNF-α (green, M1 type macrophage marker) were visualized using indirect immunostaining. The images were captured on Zeiss Meta 510 confocal microscope (Carl Zeiss).

### Flow Cytometry

MH-S cells were washed three times with PBS, and then the cells were centrifuged for 5 min in 1,500 rpm and resuspended in 500 μl of PBS. The untreated groups of cells were separately added with 0.5 μl of PBS, fluorescein isothiocyanate (FITC), and phycoerythrin (PE), which were used for calibration parameters. Other groups of cells were separately added 200 μl of CD38 antibody with PE (1:1,000 in PBS). Cells were incubated in the dark for 30 min at room temperature, washed two times with PBS, and then resuspended in 1 ml of PBS. Early growth response protein 2 (Egr2) was marked by Staining Intracellular Antigens for Flow Cytometry kit following the user guidelines provided by the company (00-5521-00, Thermo Fisher Scientific). The cells were filtered by using a 40-μm filter, and the macrophage type was tested by flow cytometry. M1 macrophages were marked by CD38 with FITC fluorescence (11038181, Thermo Fisher); and M2 macrophages were marked by early growth response protein 2 (Egr2) with PE fluorescence (12669182, Thermo Fisher) (35, 36). The percentages of M1 and M2 were recorded by flow cytometry. The ratio of M1 to M2 was calculated using the percentages of M1 divided by the percentages of M2.

### *In vivo* Imaging

C57BL/6N and SHIP knockout mice were infected with bioluminescence-generating PAO1 (Xen 41) for 12 h, anesthetized by ketamine, and then imaged under an IVIS XRII system following the company's instructions (PerkinElmer-Caliper).

### Statistical Analysis

All experiments were repeated at least three times. Data were presented as mean ± standard error of the mean (SEM). Statistical analysis was performed with GraphPad (GraphPad Software, La Jolla, CA) using one-way ANOVA plus Tukey *post hoc* test, and statistically significant differences are indicated as ^*^p < 0.05 and ^**^p < 0.01 (37).

## Results

### SHIP-1^-/-^ Mice Exhibited Increased Susceptibility and Severe Lung Injury After PAO1 Infection

**Figure 1:**
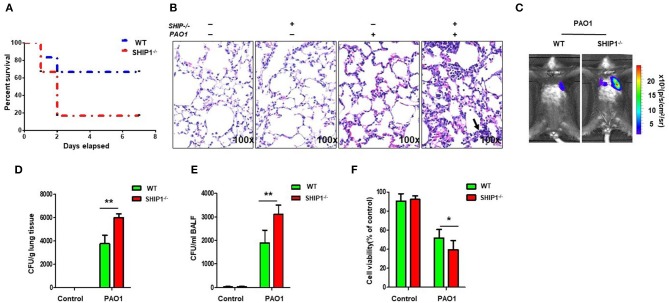
SHIP-1^−/−^ mice show increased susceptibility to PAO1 infection and severe disease. **(A)** Survival curves of SHIP-1^−/−^ and wild-type (WT) mice infected by PAO1 with 2 × 10^7^ colony-forming units (CFUs) per mouse. The survival test is represented by Kaplan–Meier survival curves (95% confidence interval, *n* = 6 for each group, *p* = 0.043). **(B)** Increased alveolar ruptures, thickened alveolar walls, and inflammatory cell infiltration were evaluated by H&E staining infected for 12 h. Arrows indicate the intensity of inflammation and disease progression. **(C)** Lung images were obtained using the bioluminescence in an IVIS XRII system after infection. Data are shown as mean ± SEM of three independent experiments. Bacterial burdens in the lungs **(D)** and bronchoalveolar lavage fluid (BALF) **(E)** were determined after PAO1 infection for 12 h. **(F)** Cell viability of AM were evaluated through the MTT assay after infection PAO1 for 2 h (data are displayed as mean ± SEM and are representative of three mice evaluated in each group, one-way ANOVA with Tukey *post hoc* test in **(D**–**F)**; *p < 0.05, **p < 0.01).

To explore the roles of SHIP-1 in PAO1 infection, we challenged SHIP-1^−/−^ and WT mice (on C57BL/6N background) with PAO1 (2 × 10^7^ CFU/mouse) and performed a survival assay (uninfected group mice as control). We found that SHIP-1^−/−^ mice exhibited markedly increased susceptibility to infection (approximately 66.7% SHIP-1^−/−^ mice died within 48 h after infection, but just 16.7% WT mice died at this time point (Figure 1A). The outcome was presented with a Kaplan–Meier survival curve (*p* = 0.043). Lung injury dramatically increased in SHIP-1^−/−^ mice compared with WT mice (Figure 1B). To better monitor the difference between SHIP-1^−/−^ and WT mice, we set out to gauge the *in vivo* dynamics of infection with a bioluminescence bacterial strain Xen 41 (Figure 1C). This strain is derived from PAO1 and showed intense bioluminescence on both a dish and an Eppendorf tube (Figure S1A). We found a susceptible phenotype in SHIP-1^−/−^ mice, compared with WT mice, with faster bacterial diffusion in the lung after instilling Xen 41 to mouse lungs for 12 h (Figure 1C). Importantly, the amount of Xen 41 in the lung and BALF were increased in the SHIP^−/−^ mice as detected by CFU assay (Figures 1D,E). Concomitantly, we isolated alveolar macrophages (AM) to detect their viability after 12-h PAO1 infection in mice (Figure 1F). The viability of AM cells that were obtained from SHIP-1^−/−^ mice was significantly reduced. Overall results suggest that SHIP-1 may be required for host defense against PAO1 infection in acute pneumonia models.

### SHIP-1 Deficiency Aggravated Lipid Aggregates, Oxidative Damage, and Bacterial Dissemination Upon PAO1 Infection

**Figure 2:**
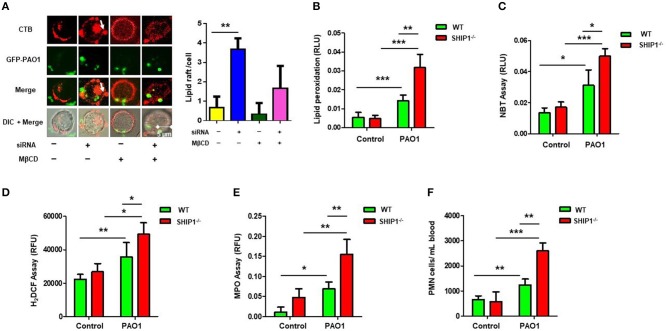
SHIP-1 deficiency aggravates lipid raft aggregation, oxidative damage, and bacterial dissemination after PAO1 infection. **(A)** MH-S cells were transfected with SHIP-1 siRNA (or negative control siRNA) for 48 h or treated with lipid raft blocker MβCD at 10 mM, then infected with green fluorescent protein (GFP)–PAO1 for 30 min, and stained with lipid raft marker (TRITC-labeled cholera toxin B, CTB-red) at 1/10,000 dilution; images were captured using microscope and assessed using ImageJ software on the basis of minimal 100 cells; arrows indicate the extent of lipid raft aggregation. **(B)** Peroxidation product was measured by a colorimetric assay in the lung tissue lysate. **(C)** Superoxide production in bronchoalveolar lavage fluid (BALF) cells significantly increased in PAO1-infected SHIP-1^−/−^ mice compared with WT mice using the nitroblue tetrazolium (NBT) assay (1 mg/ml) at 560-nm absorbance. **(D)** Oxidative stress was increased in SHIP-1^−/−^ AM cells as determined by the H_2_DCF fluorescence assay (5 μM) at 488 nm. **(E)** Myeloperoxidase (MPO) was measured from the lungs of SHIP^−/−^ mice as compared with WT mice. **(F)** Polymorphonuclear neutrophils (PMNs) were significantly increased in infected SHIP-1^−/−^ mice compared with WT mice (the data are mean ± SEM and are representative of three independent experiments, one-way ANOVA with Tukey *post hoc* test, *p < 0.05, **p < 0.01, ***p < 0.001).

We previously found that Lyn and membrane rafts were involved in phagocytosis and disease progression during *Pseudomonas aeruginosa* infection (6). To investigate the involvement of lipid rafts in the SHIP-1 model, we transfected AM cells with SHIP-1 siRNA (knocking down confirmed by western blotting) and then infected the cells with GFP–PAO1 at 20:1 of multiplicity of infection (MOI) to track lipid rafts using CTB conjugated with Alexa Fluor™ 594. Lipid rafts aggregates were also evaluated using lipid rafts blocker MβCD, which significantly disrupted lipid raft platforms. Our data showed that aggregates significantly increased in SHIP-1 siRNA cells compared with negative control cells (Figure 2A). Furthermore, WT mice and SHIP-1^−/−^ mice were anesthetized using 45 mg/kg of ketamine and instilled intranasally with 2 × 10^7^ CFUs of PAO1 for 12 h; the lipid peroxidation was measured in the lung (Figure 2B), liver (Figure S2A), and spleen (Figure S2B). The oxidation state of the tissue and increased recruitment of PMNs and MPO activity are well-recognized for indicating the endpoints of acute lung injury (38, 39). To further assess the damage of lung tissue in SHIP-1^−/−^ mice, the amount of reactive oxygen species (ROS) released and cellular superoxide was measured by using NBT (Figure 2C) and H_2_DCF assay (Figure 2D). Simultaneously, MPO activity and the amount of PMN infiltration into the blood smear were quantified using MPO Activity Assay Kit (ab105136, Cambridge, MA, USA) in the lung (Figure 2E), liver (Figure S2C), and spleen (Figure S2D) and by HEMA-3 staining assay (Figure 2F). Collectively, these data suggest that PAO1 SHIP-1 deficiency or knockdown aggregated lipid accumulation, oxidative damage, and bacterial dissemination after PAO1 infection.

### SHIP-1 Deficiency Impeded Phagocytosis of Macrophages and Induced M2 Polarization

**Figure 3:**
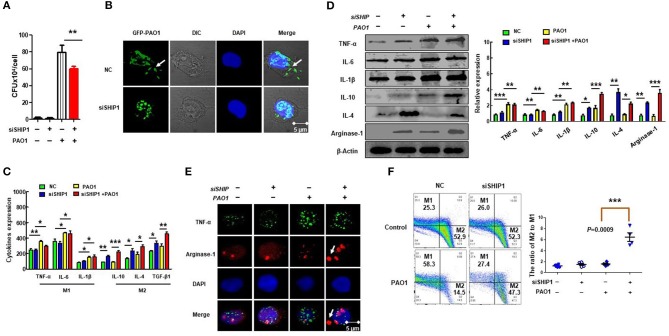
SHIP-1 deficiency disrupts phagocytosis and M2 polarization. MH-S cells were transfected with SHIP-1 siRNA or negative siRNA for 48 h, followed by PAO1 infection for 2 h (uninfected group MH-S cells as control), knockout efficiency was detected by western blotting (Figure S3). **(A)** Phagocytosis in MH-S cells was measured by colony-forming units (CFU). **(B)** The number of bacteria engulfed by macrophages was observed by immunofluorescence by using green fluorescent protein (GFP)–PAO1, which can be directly detected through confocal with a green fluorescent signal and has the same virulence as PAO1 in MH-S cells. **(C)** The expression levels of inflammatory factors related to M1 macrophage markers (IL-6, INF-γ, and TNF-α) and M2 macrophage markers (IL-10, IL-4, and TGF-β1) were analyzed by ELISA in MH-S cells. **(D)** M1 macrophage type markers (TNF-α, IL-6, and IL-1β) and M2 macrophage markers (IL-10, IL-4, and arginase-1) were analyzed by western blotting in MH-S cells. **(E)** Representative M1 macrophages marker (TNF-α) and M2 macrophages marker (arginase-1) were analyzed by immunofluorescence in MH-S cells. **(F)** The percentage of M2 macrophages to M1 macrophages was analyzed by CD38/Egr2 by flow cytometry in MH-S cells (the data are mean ± SEM and are representative of three independent experiments, one-way ANOVA with Tukey *post hoc* test, *p < 0.05, **p < 0.01, ***p < 0.001).

Macrophage phagocytosis is important for the immune response (40). To determine the impact of SHIP-1 on phagocytosis, CFU assay and immunofluorescence were performed in MH-S by using GFP–PAO1, which can be directly detected through confocal with a green fluorescent signal and has the same virulence as PAO1 (Figures 3A,B), which showed decreased phagocytosis in SHIP-1 siRNA-transfected MH-S cells. The ratio of proinflammatory (M1) to anti-inflammatory (M2) phenotypes often determines the immune response intensity (41). Appropriate polarization is essential to maintain the inflammatory balance and phagocytosis in response to different external environments (42, 43). Therefore, we believe that may be related to SHIP deficiency-induced macrophage polarization after PAO1 infection. To further analyze the role of macrophage polarization in infected SHIP-1^−/−^ mice, we assessed macrophage types by ELISA (Figure 3C), western blotting (Figure 3D), and immunofluorescence (Figure 3E) using macrophage M1 markers (TNF-α, IL-1β, and IL-6) and M2 markers (IL-10, IL-4, arginase-1, and TGF-β1). Studies have shown that the ratio of CD38 and Egr2 (early growth response protein 2) can be used to characterize types of macrophage. M1 macrophages were labeled by CD38 with FITC fluorescence, and M2 macrophages were stained by Egr2 with PE fluorescence (35). The ratio of M2/M1 was determined by flow cytometry (Figure 3F). Our data displayed that M1 phenotype inflammatory factors were elevated in PAO1-infected MH-S cells (both PAO1-infected group and SHIP deficiency's PAO1 infected group). SHIP deficiency elicited a large number of expressions of the M2 phenotype inflammatory factors. Collectively, these findings imply that SHIP-1 deficiency hampered phagocytosis of macrophages, which may be associated with M2 polarization aggravation.

### SHIP-1 Deficiency Deucedly Activated Phosphorylation of PI3K/Akt–STAT5–Trib1 Circuit After PAO1 Infection

**Figure 4:**
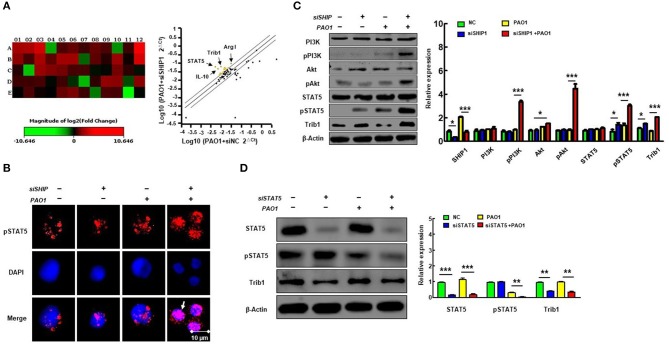
SHIP-1 deficiency elevates phosphorylation of PI3K/Akt–STAT5–Trib1 after PAO1 infection in mice. MH-S cells were transfected with SHIP-1 siRNA or negative siRNA for 48 h, followed by PAO1 infection for 2 h (uninfected group MH-S cells as control); knockout efficiency was detected by western blotting (see Figure S3). **(A)** Sixty genes associated with macrophage function and phagocytosis were screened by qPCR. Relative expression of groups was normalized by the relative expression of housekeeping gene. The ratio of the relative expression of SHIP-1 siRNA to negative siRNA was counted. Heat maps are used to indicate differential expression of genes; each color of A1–E12 represents the expression level of a single gene. The corresponding gene names and primers are listed in Supplemental Table 1; yellow dots indicate increased expression of gene, and blue dots indicate decreased expression of genes in scatter plot (more than a 2-fold change and *p* < 0.05 by Student's *t*-test). **(B)** The phosphorylated STAT5 in the nucleus was analyzed by immunofluorescence. **(C)** Western blotting shows the activated PI3K/Akt–STAT5–Trib1 signaling proteins circuit of SHIP-1 siRNA in MH-S cells compared with control. **(D)** Western blotting shows reduced expression of Trib1 in STAT5 siRNA cells compared with controls (the data are mean ± SEM and are representative of two independent experiments, Student's *t*-test in **(B)**, one-way ANOVA with Tukey *post hoc* test in **(C,D)**, *p < 0.05, **p < 0.01, ***p < 0.001).

To further characterize the critical role of SHIP-1 and macrophage polarization during bacterial infection, we performed qPCR microarray to screen the genes that are involved in macrophage function and phagocytosis after transfection of SHIP siRNA and control siRNA in PAO1 infected MH-S cells (Figure 4A) (gene primers are shown in Table S1). We found that expression of STAT5 and Trib1 was significantly increased. STAT5, a transcription factor, has been shown to be related with regulation of M2 macrophage programming (16). Trib1 is also critical for the differentiation and tissue residence of M2-like macrophages (19). Therefore, we posit that STAT5 and Tribbles homolog 1 (Trib1) may play a crucial role in SHIP deficiency-associated impairment of macrophage phagocytosis and aggravating M2 polarization. Next, phosphorylation of STAT5 nuclear translocation is determined by immunofluorescence (Figure 4B); and PI3K, STAT5, and Trib1 protein levels were analyzed by western blotting (Figure 4C). These results revealed that SHIP-1 knockdown activated nuclear transfer of phosphorylation of STAT5, indicating a PI3K/Akt–STAT5–Trib1 circuit in PAO1-infected MH-S cells. We also found that Trib1 was significantly reduced after transfecting MH-S cells with STAT5 siRNA (Figure 4D). These findings demonstrate that SHIP-1 knockdown induced macrophage polarization, which is likely to be regulated by the PI3K/Akt–STAT5–Trib1 signal pathway.

### Suppression of STAT5 Expression Reduced Polarization of M2 Macrophages and Impaired Phagocytosis

**Figure 5:**
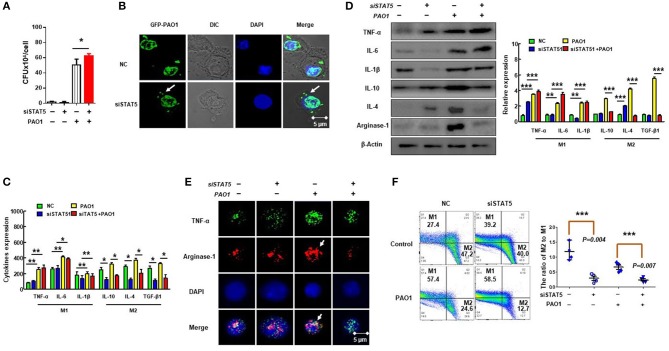
Suppression of STAT5 expression improves polarization of M2 macrophages to impair phagocytosis. MH-S cells were transfected with STAT5 siRNA or negative siRNA for 48 h, followed by PAO1 infection for 2 h (uninfected group MH-S cells as control); knockout efficiency was detected by western blotting (see Figure S3). **(A)** Phagocytosis by MH-S was measured by colony-forming unit (CFU). **(B)** Immunofluorescence was performed to reveal the number of green fluorescent protein (GFP)–PAO1 engulfed by macrophages. **(C)** Markers of M1 macrophages (IL-6, INF-γ, and TNF-α) and M2 macrophages (IL-10, IL-4, and TGF-β1) were analyzed by ELISA. **(D)** M1 macrophage markers (TNF-α, IL-6, and IL-1β) and M2 macrophage markers (IL-10, IL-4, and arginase-1) were quantified by western blotting. **(E)** Typical marker for M1 (TNF-α) and M2 (arginase-1) was analyzed by immunofluorescence. **(F)** The ratio of M2 macrophages to M1 macrophages was measured using CD38 and Egr2 antibody by flow cytometry (the data are mean ± SEM and are representative of three independent experiments, one-way ANOVA with Tukey *post hoc* test, *p < 0.05, **p < 0.01, ***p < 0.001).

Previous studies have shown that M2 macrophage polarization is related to SHIP-1 deficiency and activation of STAT5 (16). To verify whether M2 macrophage polarization was regulated *via* activated STAT5, we evaluated the phagocytosis of macrophages by CFU and immunofluorescence after suppression of STAT5 expression using RNAi (Figures 5A,B) and found that MH-S cells with STAT5 siRNA transfection had increased phagocytosis. Moreover, the polarization of M2 macrophages was significantly decreased in STAT5 siRNA-transfected MH-S cells with PAO1 infection as measured by ELISA (Figure 5C), western blotting (Figure 5D), immunofluorescence (Figure 5E), and flow cytometry (Figure 5F). These results indicated that suppression of STAT5 expression reversed the over-activated M2 macrophage polarization and impaired phagocytosis that was mediated by hyperactivation of the PI3K/Akt–STAT5–Trib1 signal pathway due to SHIP-1 deficiency. Consistently, these findings demonstrate that activated STAT5 is involved in the process of M2 macrophage polarization.

### Trib1 Played Critical Roles in Polarization of M2-Like Macrophages and Phagocytosis

**Figure 6:**
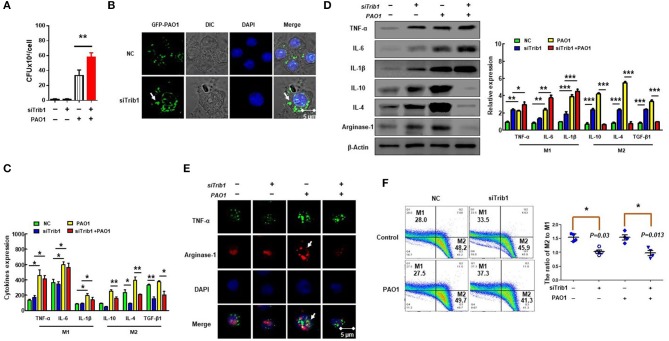
Trib1 regulates polarization of M2-like macrophages. MH-S cells were transfected with Trib1 siRNA or negative siRNA for 48 h, followed by PAO1 infection for 2 h (uninfected group MH-S cells as control); knockout efficiency was detected by western blotting (Figure S3). **(A)** Phagocytosis was measured by colony-forming unit (CFU). **(B)** Immunofluorescence was used to show the number of intracellular green fluorescent protein (GFP)–PAO1. **(C)** ELISA determining the expression of M1 macrophage markers (IL-6, INF-γ, and TNF-α) and M2 macrophage markers (IL-10, IL-4, and TGF-β1). **(D)** Western blotting analyzing the expression of M1 macrophage markers (TNF-α, IL-6, and IL-1β) and M2 macrophages markers (IL-10, IL-4, and arginase-1). **(E)** TNF-α for M1 macrophage marker and arginase-1 for M2 macrophage marker analyzed by immunofluorescence. **(F)** The relative expression levels of M1 macrophages (CD38) and M2 macrophages (Egr2) were detected by flow cytometry. (The data are mean ± SEM and are representative of three independent experiments, one-way ANOVA with Tukey *post hoc* test, *p < 0.05, **p < 0.01, ***p < 0.01).

**Figure 7:**
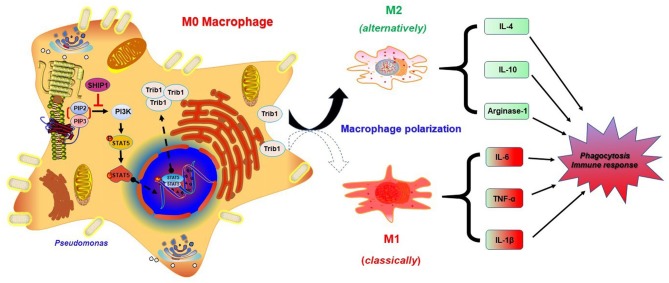
Schematic illustration of the signaling pathways for SHIP-1 regulating phagocytosis and M2 polarization through the Akt–STAT5–Trib1 circuit. Infected macrophages inhibit the overexpression of PI3K by SHIP-1, preventing hyperactivation of STAT5, and Trib1, which is critical for maintaining the polarization balance of macrophages and resistance to bacteria by secreting diverse inflammatory factors and regulating phagocytosis. Depletion of SHIP-1 triggers the excessive activation of PI3K/Akt–STAT5–Trib1 signal pathway, causing macrophage polarization imbalance to superabundant switch to M2. M1 macrophage markers (TNF-α, IL-6, and IL-1β) and M2 macrophage markers (IL-10, IL-4, and arginase-1) were used to evaluate macrophage polarization. Both defect in phagocytosis and dysregulated cytokine production may cause the aggravated infection. Ultimately, impaired phagocytosis of macrophages leads to the expansion of infection.

Trib1 has the potential to be regulated by MAPK and Akt signaling because it contains a unique pseudokinase C-tail, which can be targeted by MAPKK/MEK family. Importantly, it also contains a pseudokinase domain that can be modulated by Akt signaling (44). Research has shown that Trib1-deficient (Trib1^−/−^) mice exhibited elevated phagocytic capacity and reduced M2-polarized macrophages in bone marrow-derived macrophages (BMDMs) (21). To clarify whether the excessive activation of Trib1 influences macrophage function, we evaluated the phagocytosis of macrophages by CFU (Figure 6A) and immunofluorescence (Figure 6B) after transfection of Trib1 siRNA into MH-S cells. We found that the phagocytosis of macrophages was increased after Trib1 RNAi. Furthermore, M1 and M2 macrophage-related markers were determined by ELISA (Figure 6C), western blotting (Figure 6D), immunofluorescence (Figure 6E), and flow cytometry (Figure 6F). Consistently, our data indicate that suppression of Trib1 expression reversed the over-activated M2 macrophage polarization and impaired phagocytosis that was mediated by hyperactivation of the PI3K/Akt–STAT5–Trib1 signal pathway due to SHIP-1 deficiency. Collectively, these data strongly support that SHIP-1 regulates phagocytosis and M2 polarization through the PI3K/Akt–STAT5–Trib1 circuit (Figure 7).

## Discussion

In this study, we investigated the role of SHIP-1 in PAO1 infection using SHIP-1^−/−^ mice, which showed increased susceptibility and worsened infectious disease. Similarly, SHIP-1 deficiency aggravated lipid raft aggregates, oxidative damage, and bacterial dissemination. Importantly, our data elaborated a new mechanism that SHIP-1 deficiency activates PI3K/Akt–STAT5–Trib1 signal pathway to regulate phagocytosis of macrophages and promote M2-like polarization.

Previous studies have shown that SHIP-1 is an important regulator of immune cell function through the negative regulation of the PI3K–Akt pathway (45). SHIP-1^−/−^ mice exhibited a myeloproliferative response, indicating that an increase of macrophages or monocytes was due to enhanced proliferation and survival of their progenitors (25, 46). SHIP-1^−/−^ mice may have spontaneous inflammation in the lung, suggesting that SHIP-1 may be an important negative regulator of inflammatory responses (47). We evaluated the survival of SHIP-1^−/−^ mice and lung injury. Our results demonstrate that SHIP-1^−/−^ mice showed increased susceptibility and severe lung injury after PAO1 infection. These results indicate that SHIP-1 is essential for resistance to bacterial infection. Lipid rafts play important roles in innate and adaptive immunity by recruiting cellular signaling modifiers for T-cell activation, phagocytosis, and so on (48). The integrity of lipid rafts is implicated to be essential for LPS cellular activation owing to the presence of CD14, chemokine receptor 4, and toll-like receptor 4 on lipid microdomains of cell membrane following LPS stimulation (49). LPS stimulation can lead to lipid raft mobilization around SHIP-1, followed by activation of MAPK and release of TNF-α (50). Although the regulation of PI3K-Akt is not fully understood, our study reveals that gram-negative bacterial-mediated SHIP-1 mobilization to lipid rafts is related to PI3K-Akt (50, 51). Lipid rafts dynamic refers to the microdomain formation when membrane lipids and proteins intermingle, which may be a signal for cells to respond to acute infections. Our results showed that SHIP-1-deficient mice exhibited faster bacterial dissemination. Moreover, the penetration of PMN is increased in the lungs and increased ROS secretion that aggravates lung injury. Collectively, these observations indicate that SHIP-1 is critically involved in host inflammatory responses in *Pseudomonas aeruginosa* infection.

Macrophages are a type of white blood cells located in tissues derived from monocyte (52). Their main function is to phagocytize pathogens and activate lymphocytes or other immune cells to respond to pathogens, which is vital for innate immunity and adaptive immunity (53). Macrophages are divided into two subtypes according to their functions: proinflammatory M1 macrophages and anti-inflammatory M2 macrophages (54). The function of macrophages is regulated by multiple signaling pathways (55). Macrophage polarization is a process to adapt different functional programs in response to the signals from their microenvironment (42). We revealed that SHIP-1^−/−^ mice displayed skewed M2 macrophages, which are hyporesponsive to proinflammatory stimuli and associated with tissue remodeling, wound healing, Th2 immunity promotion, and debris clearing, indicating that SHIP-1 is a negative regulator of M2 (56). SHIP-1^−/−^ mice have the tendency to spontaneously develop chronic lung diseases with myeloid cell infiltration and macrophage subpopulation accumulation, and the number of M2 usually increased after infection (57, 58). However, the molecular mechanism of SHIP-1-mediated macrophage polarization during *P. aeruginosa* infection is still largely unclear. We evaluated the role of macrophage polarization and phagocytosis in SHIP-1 knockdown MH-S cells, and our results demonstrated that SHIP-1 deficiency impaired phagocytosis and M2 polarization. Trib1 proteins have also been reported to regulate the inflammation and macrophage polarization by activating MAPK (59, 60). We found that Trib1 is critically involved in macrophage polarization and phagocytosis by suppression of Trib1 expression in MH-S cells. These results indicate that SHIP-1 regulates M2 macrophage polarization and phagocytic function by activating the Akt–STAT5–Trib1 axis.

In summary, we have investigated the role of SHIP-1 in PAO1-challenged SHIP-1^−/−^ mice and identified SHIP-1 as a negative regulator of inflammatory responses through a PI3K/Akt–STAT5–Trib1 circuit. The aggravated infectious phenotype is involved in macrophage polarization and phagocytosis following SHIP-1 deletion. Overall, this particular phenotype results from release of various proinflammatory cytokines that can be inhibited by SHIP-1. Mechanistically, SHIP-1 deficiency activates phosphorylation of PI3K and regulates STAT5 nuclear transcription to induce the expression of Trib1. These findings reveal that SHIP-1 plays important roles in regulating inflammatory responses and macrophage function during *P. aeruginosa* infection through the PI3K/Akt–STAT5–Trib1 axis. Our research is the first demonstration that SHIP can indirectly regulate the expression of Trib1 through altering STAT5 activation to mediate the function of macrophages, which may provide significant insight into development of novel therapeutics to conquer bacterial infection. These studies may have some limitations, as we have not gone back to investigate the M1/M2 polarization in cell-specific manners in SHIP-1^−/−^ mice. In addition, studies with additional time points besides 12 (mice) and 2 h (cells) would provide holistic and dynamic mechanisms for strengthening our newly discovered role of SHIP-1. Hence, further study is warranted to understand how the M2 polarization caused by the SHIP deficiency in animals will affect the susceptibility to this bacterium in SHIP-1^−/−^ mice.

## Data Availability Statement

The datasets generated for this study are available on request to the corresponding author.

## Ethics Statement

The animal study was reviewed and approved by University of North Dakota Institutional Animal Care and Use Committee (IACUC).

## Author Contributions

SQ, YZ, HW, JL, and MW designed the project and wrote the manuscript. SQ, CZ, BP, HW, XD, and SQ designed and performed most of the experiments. JS, YZ, HW, MW, XD, ZX, and SQ analyzed the data.

### Conflict of Interest

The authors declare that the research was conducted in the absence of any commercial or financial relationships that could be construed as a potential conflict of interest.
