# Supplementary material for: SHIP-1 Regulates Phagocytosis and M2 Polarization Through the PI3K/Akt–STAT5–Trib1 Circuit in Pseudomonas aeruginosa Infection
Source: Front Immunol. 2020 Mar 18;11:307. doi: 10.3389/fimmu.2020.00307 (PMC7093384; doi:10.3389/fimmu.2020.00307)
Supplement: Supplementary file 2 [file Data_Sheet_1.pdf]

Supplementary Material

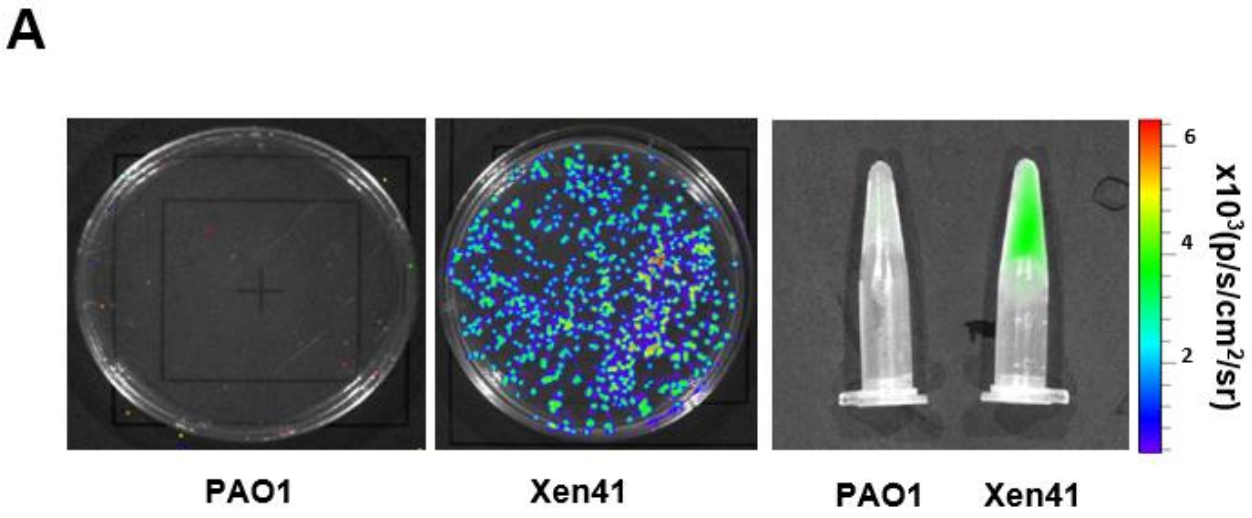

Figure S1. Analyzing bacterial bioluminescence. (**A**) Bioluminescence of Xen41 bacteria grown on a petri-dish (Left) and an Eppendorf tube (right). Image was obtained using IVIS XRII system.

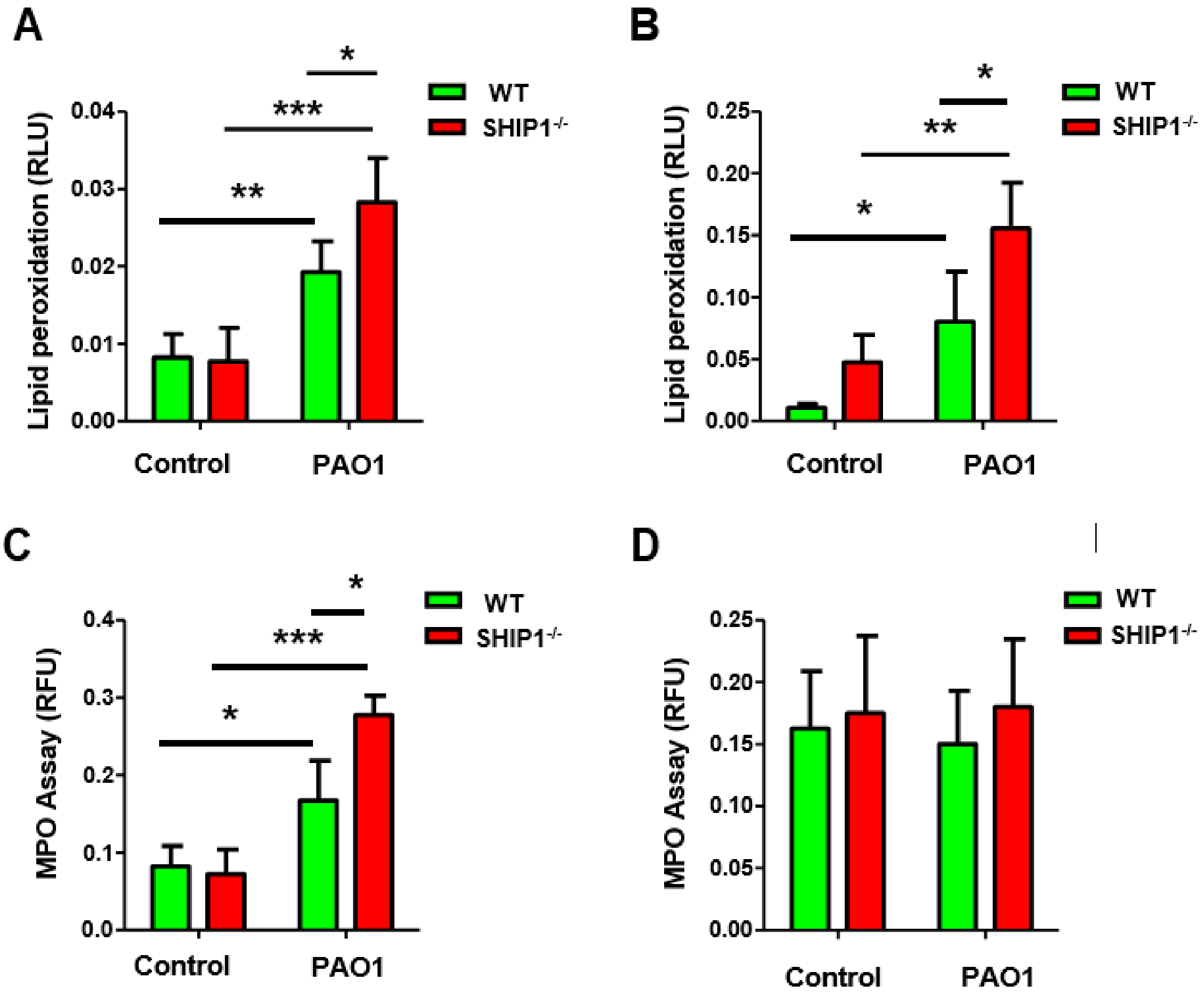

Figure S2. SHIP1 deficiency aggravated oxidative damage upon Pa infection. Peroxidation product was measured by a colorimetric assay (Calbiochem) according to the manufacturer's instructions in the liver (**A**) and kidney (**B**). Elevated activity of MPO was observed in the liver (**C**) and the kidney (**D**). Absorbance was readat 460 nm at 2 minutes intervals. (The data are means ±SEM and are representative of three independent experiments, student One Way ANOVA plus Tukey post hoc test, statistically significant differences are indicated as **P<0.05, **P<0.01*).

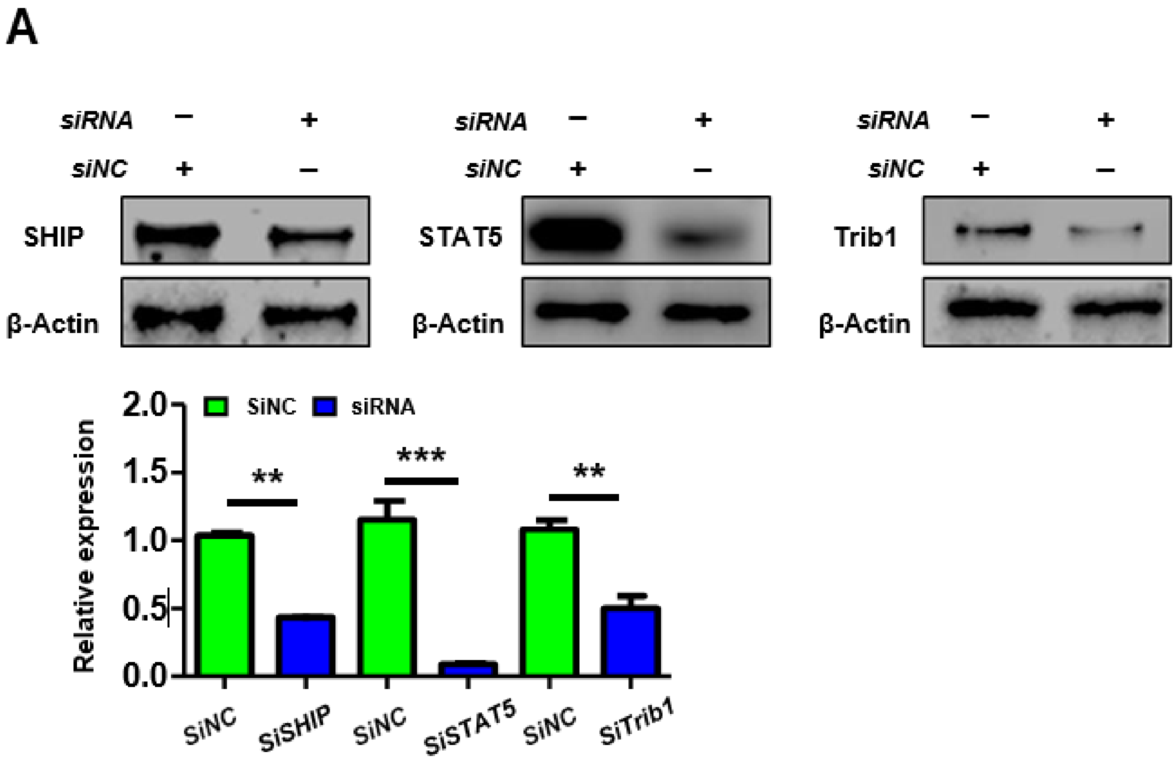

Figure S3. RNAi effectively knocks down the expression of the protein. Knockout efficiency was detected by western blotting **(A)**. (The data are means ± SEM and are representative of two independent experiments, Student’s t test, statistically significant differences are indicated as **P<0.05*, ***P<0.01*).
